# A Rare Class of New Dimeric Naphthoquinones from *Diospyros lotus* have Multidrug Reversal and Antiproliferative Effects

**DOI:** 10.3389/fphar.2015.00293

**Published:** 2015-12-16

**Authors:** Abdur Rauf, Ghias Uddin, Bina S. Siddiqui, Joseph Molnár, Ákos Csonka, Bashir Ahmad, Diana Szabó, Umar Farooq, Ajmal Khan

**Affiliations:** ^1^Department of Geology, University of SwabiSwabi, Pakistan; ^2^Institute of Chemical Sciences, University of PeshawarPeshawar, Pakistan; ^3^H.E.J. Research Institute of Chemistry, International Center for Chemical and Biological Sciences, University of KarachiKarachi, Pakistan; ^4^Department of Medical Microbiology and Immunobiology, Faculty of Medicine, University of SzegedSzeged, Hungary; ^5^Center of Biotechnology and Microbiology, University of PeshawarPeshawar, Pakistan; ^6^Department of Chemistry, COMSATS Institute of Information TechnologyAbbottabad, Pakistan

**Keywords:** *Diospyros lotus*, naphthoquinones, *P*-glycoprotein, human *ABCB1* gene transfected mouse T-cell lymphoma, antiproliferative, MDR

## Abstract

Three new dimeric naphthoquinones, 5,4′-dihydroxy-1′-methoxy-6,6′-dimethyl-7,3′-binaphthyl-1,4,5′,8′-tetraone (**1**), 5′,8′-dihydroxy-5-methoxy-6,6′-dimethyl-7,3′-binaphthyl-1,4,1′,4′-tetraone (**2**) and 8,5′,8′-trihydroxy-6,6′-dimethyl-7,3′-binaphthyl-1,4,1′,4′-tetraone (**3**), were isolated from the roots of *Diospyros lotus.* Their structures were elucidated by spectroscopic techniques, including 1D and 2D NMR, such as HSQC, HMBS, NOESY, and *J*-resolved. Compounds **1–3** were evaluated for their effects on the reversion of multidrug resistance (MDR) mediated by *P*-glycoprotein through use of the rhodamine-123 exclusion screening test on human *ABCB1* gene transfected L5178Y mouse T-cell lymphoma. Compounds **1–3** were also assessed for their antiproliferative and cytotoxic effects on L5178 and L5178Y mouse T-cell lymphoma lines. Both **1** and **2** exhibited promising antiproliferative and MDR-reversing effects in a dose-dependent manner. The effects of the tested compounds on the activity of doxorubicin were observed to vary from slight antagonism to antagonism.

## Introduction

The *Diospyros* genus in the Ebenaceae family consists of about 500 species. This genus is widely distributed in tropical and subtropical regions throughout the world and is native to the Himalayan region (Uddin et al., 2011). *Diospyros lotus* grows up to 9 m in height in semi-shaded areas (Uddin et al., 2014). *Diospyros* species are known for their multiple uses in therapeutic and folk medicine. Different parts of this plant are used for different diseases: the leaves are used to treat lumbago, the fruits as a carminative to cure biliousness, the seeds as a sedative, and the bark as an astringent and febrifuge (Rauf et al., 2014). A leaf extract of *D. kaki* (Japanese persimmon) in combination with jasmine is used in anti-tobacco smoking candies (Uddin et al., 2013). Various triterpenoids of the lupane, oleanane, and ursane series have been isolated and proved to exhibit anti-inflammatory activity (Uddin et al., 2013). *Diospyros* species are used as traditional medicines, e.g., as an antifungal, to treat hiccough, for internal hemorrhage, for bedwetting in children, as a woman’s drug for insomnia, as an antihypertensive, to treat dyspnea, as a vermicide and vermifuge, as a sedative, as an antifebrile, and as a bactericide (Tezuka et al., 1973; Ganapaty et al., 2006). A *D.* *lotus* extract and isolated compounds have been reported to display promising antiproliferative activity (Loizzo et al., 2009).

Quinone moieties are present in many drugs, such as anthracyclines, daunorubicin, doxorubicin, mitomycin, mitoxantrones, and saintopin, which are used clinically in the therapy of solid cancer (Verma, 2006). Moreover, some naphthoquinones isolated from *Diospyros* species, such as plumbagin, exert cytotoxic activity (Padhye et al., 2012).

Multidrug resistance (MDR) is the main clinical challenge for the active treatment of cancer (chemotherapy; Szabo and Molnar, 1997). There are numerous mechanisms by which tumor cells develop resistance to cytotoxic secondary metabolites. One of them is produced by the overexpression of ATP-binding cassette (ABC) proteins or breast cancer resistance protein (BCRP). The ATP-binding cassette transporters represent the largest family of transmembrane proteins that bind ATP and use the energy to drive the transport of various molecules across cell membranes (Gottesman and Ambudkar, 2001; Leonard et al., 2003). ABC eﬄux transporters extrude a broad range of amphiphilic compounds against the concentration gradient in an energy-dependent fashion. Many of the ABC transporters have dedicated physiological functions, and afford normal tissue protection in the brain vessels, liver, and kidney (Gottesman et al., 2002; Sarkadi et al., 2006; Szakacs et al., 2006).

The firstly identified drug eﬄux protein was the *P*-glycoprotein (*P*-gp, MDR1, ABCB1), encoded by the *ABCB1* gene. *P*-gp is composed of 1280 amino acids (170 kDa) organized in two transmembrane domains (Szakacs et al., 2006). This protein is overexpressed in several human tumors and can extrude a wide range of drugs (anticancer, antibiotics, antidepressants, antihistamines, antiarrhythmics, immunosuppressants, HIV protease inhibitors and steroids). Many drug molecules, such as tamoxifen, valspodar, dexniguldipine, and tariquidar, have been proposed to suppress the action of *P*-gp (Germann et al., 1993; Lopez and Martinez-Luis, 2014).

A second cellular pump is multidrug-resistant protein 1 (MRP1, ABCC1), described in Cole et al. (1992). MRP1 is an eﬄux pump originally discovered in doxorubicin-resistant lung carcinoma cells displaying a multidrug resistant phenotype without ABCB1 expression.

MRP1 is expressed ubiquitously in higher levels at the blood–brain barrier, in the intestines and in the oral mucosa (He et al., 2011). MRP1 expression is higher in the lungs than in any other organ and it may have protective roles against air pollution and inhaled toxins (Sakamoto et al., 2013). The physiological substrates of MRP1 include bile acids, folic acid, leukotriene C4, and glutathione conjugates, and it confers resistance to vincristine, methotrexate, doxorubicin, and etoposide (Cole and Deeley, 2006; Li et al., 2008).

A third cellular pump type is BCRP, first cloned in the drug-resistant breast cancer cell line MCF-7 (Doyle et al., 1998). BCRP is a half-transporter member of the ABCG subfamily (ABCG2) with a size of 72 kDa. BCRP probably functions as a homodimer. The expression of BCRP overlaps largely with that of *P*-gp, because the protein can be found in tissues such as the placenta, prostate, small intestine, brain, colon, liver, and ovary (Doyle et al., 1998). Overexpression of BCRP is associated with resistance to a wide range of different anticancer agents: anthracyclines, mitoxantrone, flavopiridol, camptothecins, and antifolates (Assaraf, 2006; Bihorel et al., 2007; Robey et al., 2007).

Several studies have demonstrated the frequent occurrence of drug eﬄux proteins in cancer tissue. Some authors have reported significant correlations between the overexpression of *P*-gp or MRP-1 and a poor treatment response in solid tumors and some leukemias (Brinkhuis et al., 2002; Diestra et al., 2003; Larkin et al., 2004; Damiani et al., 2006), and a prognostic significance for BCRP overexpression in specific forms of leukemia (Larkin et al., 2004).

The current study deals with the isolation of three new dimeric naphthoquinones (**1–3**) from the chloroform (CHCl_3_) fraction of *D. lotus.* The isolated compounds (**1–3**) were evaluated for their effects on the reversion of MDR in mouse lymphoma and for their antiproliferative and cytotoxic effects on the L5178 and L5178Y mouse T-cell lymphoma cell lines. A combination assay was also applied to study the effects of the drug interactions between the dimeric naphthoquinone derivatives and the chemotherapeutic drug doxorubicin on the MDR mouse lymphoma cell line.

## Materials and Methods

### General Procedure

Melting points of compounds (**1–3**) were determined on a Bicote melting point apparatus and are uncorrected. UV-visible spectra were recorded on a Hitachi-U-3200 spectrometer, IR spectra on an FT-IR instrument (Nicolet 380), UV-visible spectra on a Shimadzu spectrometer, and ^1^H-NMR (500 MHz), ^13^C-NMR (600 MHz), HMBC (600 MHz) and HSQC (600 MHz spectra on an AVANCE AV-600 Cryoprob NMR instrument in CDCl_3_. HR-EI-MS spectra were measured on a JEOL JMS 600H mass spectrometer; EI source 70 eV. Normal-phase column chromatography (CC) was performed by using silica gel (Merck). TLC was run on pre-coated aluminum plates with silica gel 60 (F254; Merck).

### Plant Material

*Diospyros lotus* roots were collected from Razagram (Khall), Dir, KPK, Pakistan, in May 2009. The sample was authenticated by Dr. Abdur Rashid, Department of Botany, University of Peshawar, Pakistan. A voucher specimen [Bot. 20036(PUP)] has been deposited at the Herbarium, Department of Botany, University of Peshawar, Pakistan.

### Extraction and Isolation

Shade-dried roots of *D. lotus* (14 kg) were powdered and repeatedly extracted with methanol (MeOH; 64 L) at room temperature. The extracts were combined and concentrated by evaporating the solvent in a rotary evaporator under reduced pressure at a temperature below 40°C to obtain a dark red residue (202 g). This was suspended in water and successively partitioned into *n*-hexane, CHCl_3_, ethyl acetate (EtOAc), and *n*-butanol (*n*-BuOH) to afford *n*-hexane (30 g), CHCl_3_ (88 g), EtOAc (20 g), and *n*-BuOH (50 g) fractions. The CHCl_3_ fraction F-1 (30 g) was subjected to CC on silica gel (Merck silica gel 60 (0.063–0.200 mm), 5 cm × 60 cm). The column was eluted with *n*-hexane-EtOAc (100:0 → 0:100) as solvent system. A total of 105 fractions, (RF-1 to RF-105) were obtained on the basis of the TLC profiles. Fractions RF-1 to RF-10 were combined on the basis of TLC to obtain subfraction SF-1 (2 g), which was further re-subjected to CC with *n*-hexane elution to yield a reddish residue of fatty acids, while fractions RF-11 to RF-105 were combined on the basis of the TLC profiles to give major subfractions SF-3 (9.89 g) and SF-4 (3.98 g).

**FIGURE 1:**
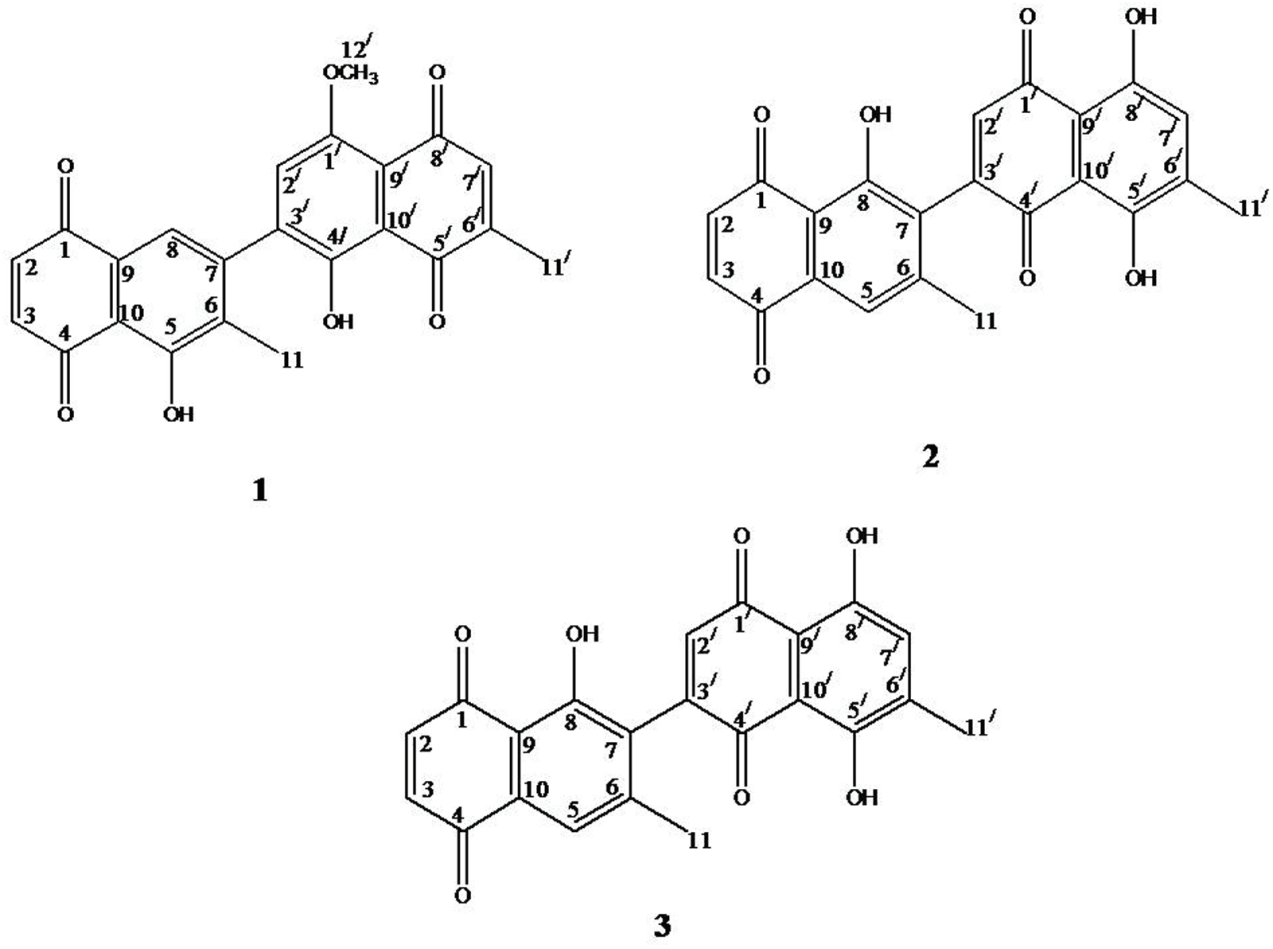
**Structures of compounds 1–3**.

Fraction SF-4 (9.89 g) was subjected to CC with *n*-hexane-EtOAc (100:0 → 10:15) elution and 60 fractions were obtained and combined on the basis of the TLC profiles, yielded two major fractions MF-1 (5.44 g) and MF-2 (3.41 g). Fraction MF-1 (5.44 g) was subjected to preparative TLC silica gel (Merck silica gel 60 F_254_), using *n*-hexane-EtOAc (85:15, RF: 0.45; 84:16, RF: 0.46; 80:20, RF: 0.47), which furnished three new dimeric naphthoquinones: 5,4′-dihydroxy-1′-methoxy-6,6′-dimethyl-7,3′-binaphthyl-1,4,5′,8′-tetraone (**1**), 5′,8′-dihydroxy-5-methoxy-6,6′-dimethyl-7,3′-binaphthyl-1,4,1′,4′-tetraone (**2**) and 8,5′,8′-trihydroxy-6,6′-dimethyl-7,3′-binaphthyl-1,4,1′,4′-tetraone (**3**) (**Figure 1**). The structure elucidation of these new compounds was based on spectroscopic analysis, including 1D and 2D NMR.

### Anticancer Assays

#### Cell Cultures

L5178 mouse T-cell lymphoma cells (ECACC cat. no. 87111908, U.S. FDA, Silver Spring, MD, USA) were transfected with pHa MDR1/A retrovirus, as previously described (Cornwell et al., 1987; Bak et al., 1989). The MDR1-expressing cell line L5178Y was selected by culturing the infected cells with colchicine. L5178 (parent) mouse T-cell lymphoma cells and the human MDR1-transfected subline were cultured in McCoy’s 5A medium supplemented with 10% heat-inactivated horse serum, 200 mM L-glutamine, and a penicillin-streptomycin mixture in 100 U/L and 10 mg/L concentrations, respectively. MDR was detected with a monoclonal antibody (Bak et al., 1989; Molnar et al., 1999). The cell lines were incubated in a humidified atmosphere (5% CO_2_, 95% air) at 37°C. The L5178 mouse T-cell lymphoma cells (PAR; ECACC Cat. No. 87111908, obtained from FDA, Silver Spring, MD, USA) were transfected with pHa MDR1/A retrovirus, as previously described by Cornwell et al. (1987). The ABCB1-expressing cell line L5178Y (MDR) was selected by culturing the infected cells with colchicine (Cornwell et al., 1987). L5178 (parent) mouse T-cell lymphoma cells and the L5178Y human ABCB1-transfected subline were cultured in McCoy’s 5A medium supplemented with 10% heat-inactivated horse serum, 200 mM L-glutamine and a penicillin-streptomycin mixture in concentrations of 100 U/L and 10 mg/L, respectively.

### Antiproliferative Assays

The antiproliferative effects of compounds **1–3** were determined in 96-well flat-bottomed microtiter plates (Molnar et al., 2004b; Gyemant et al., 2005). The antiproliferative potentials of the compounds were tested at a concentration of 1 μg/mL, using the L5178Y mouse T-cell lymphoma MDR cell line in the experimental model. The cells were distributed into 96-well flat-bottomed microtiter plates at a concentration of 100 μL in McCoy’s 5A or RPMI-1640 medium. For the antiproliferative assay, 6 × 10^3^ mouse T-cell lymphoma cells in 100 μL of medium were added to each well. The culture plates were further incubated at 37°C for 72 h for the antiproliferative effect assay. At the end of the incubation period, 20 μL of 3-[4,5-dimethylthiazol-2-yl]-2,5-diphenyltetrazolium bromide (MTT; Sigma, St. Louis, MO, USA) solution (from a 5 mg/mL stock) was added to each well. After 4 h, 100 μL of 10% sodium dodecyl sulfate (SDS; Sigma) in 0.01 M HCl was added to each well. The culture plates were further incubated at 37°C overnight. The cell growth was determined by measuring the optical density (OD) at 550 nm (ref. 630 nm) with a Multiscan EX ELISA reader (Thermo Labsystem, Cheshire, WA, USA). In the assay, the solvent did not have any effect on the cell growth at the concentrations used for half-maximal inhibitory concentration (IC_50_) calculations. IC_50_ values and the standard error of the mean (SEM) of triplicate experiments were calculated by using GraphPad Prism software version 5.00 for Windows with non-linear regression curve fitting (GraphPad Software, San Diego, CA, USA^1^).

### Assays for Cytotoxic Effects

The effects of increasing concentrations of the drugs alone on cell growth were tested in 96-well flat-bottomed microtiter plates. The compounds were diluted in 100 μL of medium. 1 × 10^4^ mouse T-cell lymphoma cells (PAR or MDR) in 50 μL of medium were then added to each well, with the exception of the medium control wells. The culture plates were further incubated at 37°C for 24 h; at the end of the incubation period, 15 μL of MTT solution (from a 5 mg/mL stock) was added to each well. After incubation at 37°C for 4 h, 100 μL of SDS solution (10% in 0.01 M HCI) was added to each well and the plates were further incubated at 37°C overnight. The cell growth was determined by measuring the OD at 540 nm (ref. 630 nm) with a Multiscan EX ELISA reader (Thermo Labsystems, Cheshire, WA, USA). In the assay, the solvent did not have any effect on the cell growth at the concentrations used for IC_50_ calculations. IC_50_ values and the SEM of triplicate experiments were calculated by using GraphPad Prism software version 5.00 for Windows with non-linear regression curve fitting (GraphPad Software, San Diego, CA, USA^2^).

### Assays for Reversal of MDR in Mouse Lymphoma Cells

The L5178Y MDR and L5178 parent cell lines were grown in McCoy’s 5A medium containing 10% heat-inactivated horse serum, supplemented with L-glutamine and antibiotics. The cells were adjusted to a density of 2 × 10^6^ mL, resuspended in serum-free McCoy’s 5A medium and distributed in 0.5 mL aliquots into Eppendorf centrifuge tubes. The tested compounds were added at 0.1–1 μg/mL final concentrations, and the samples were incubated for 10 min at room temperature. Verapamil was applied as positive control (Cornwell et al., 1987) in 10 μg/mL concentration. Next, 10 μL (5.2 μM final concentration) of the indicator rhodamine 123 (Sigma, St Louis, MO, USA) was added to the samples and the cells were incubated for a further 20 min at 37°C, washed twice and resuspended in 0.5 mL of PBS for analysis. The fluorescence of the cell population was measured with a Partec CyFlow flow cytometer (Munster, Germany). Verapamil was used as a positive control in the rhodamine 123 exclusion experiments (Gruber et al., 1988). The tested compounds were dissolved in DMSO, which was also used as solvent control. The percentage mean fluorescence intensity was calculated for the treated MDR and parental cell lines as compared with the untreated cells. The activity ratio was calculated via the following equation (Molnar et al., 2004a) on the basis of the measured fluorescence values:

$$\text{FAR}=\frac{MDR_{\text{treated}}/MDR_{\text{control}}}{parental_{\text{treated}}/parental_{\text{control}}}$$

The results presented were obtained from a representative flow cytometric experiment in which 10^5^ individual cells of the population were evaluated for the amount of rhodamine 123 retained. The data were analyzed with FlowJo software^3^.

### Checkerboard Combination Assays

A checkerboard microplate method was applied to study the effects of the drug interactions between the dimeric naphthoquinone derivatives and the chemotherapeutic drug doxorubicin on the MDR mouse lymphoma cell line. The dilutions of doxorubicin were made in a horizontal direction in 100 μL, and the dilutions of the dimeric naphthoquinone compounds were made vertically in the microtiter plate in 50 μL. The cells were resuspended in culture medium and distributed into each well in 50 μL containing 6 × 10^3^ PC3 MDR mouse T-cell lymphoma cells. The plates were incubated for 72 h at 37°C in a CO_2_ incubator. The cell growth rate was determined after MTT staining, as described above. The combination index (CI) values at 50% growth inhibition (ED_50_) were determined by using CompuSyn software^4^ (ComboSyn, Inc., Paramus, NJ. USA) to plot 4 or 5 data points for each ratio. CI values were calculated by means of the median-effect equation (Chou and Martin, 2005), where CI < 1, CI = 1, and CI > 1 represent synergism, an additive effect (or no interaction) and antagonism, respectively.

## Results and Discussion

The whole MeOH extract was suspended in water and successively partitioned with *n*-hexane, CHCl_3_, EtOAc, and *n*-BuOH. The CHCl_3_ fraction was selected for phytochemical investigation due to the presence of a greater amount of compounds as indicated by the TLC profile. CC of the CHCl_3_ fraction (30 g) on silica gel resulted in the isolation of three new dimeric naphthoquinones (**1–3**). The structures of these compounds were elucidated by spectroscopic techniques and comparisons with literature data.

Compound **1** was isolated as a yellow amorphous powder. It exhibited the molecular ion peak at *m/z*; 404.2100 a.m.u. (calcd. 404.2101) corresponding to the molecular formula C_23_H_16_O_7._ The IR spectrum showed absorption bands at 3550 cm^-1^ for OH stretching, 2924 cm^-1^ for CH stretching, 1643 cm^-1^ for CO stretching and 1634, 160, and 1460 cm^-1^ for CH aromatic stretching. The UV spectrum exhibited absorptions at 253, 296, and 435 nm.

**FIGURE 2:**
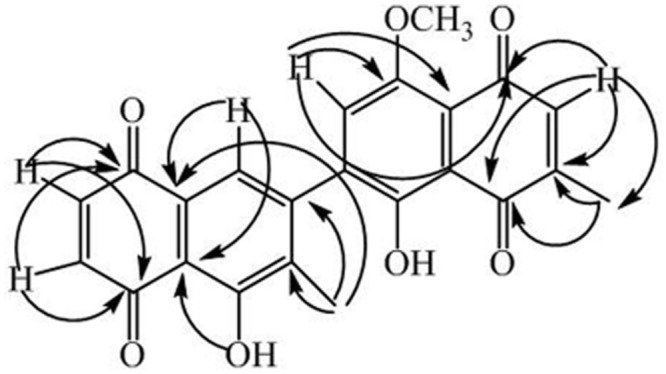
**Key HBMC correlations of compound 1**.

1D and 2D NMR studies were carried out to elucidate the structure of the compound. The assignments of protons and carbons were carried out by HMBC, HMQC, ^1^H-^1^H-COSY and *J*-resolved experiments (**Table 1**). The ^1^H-NMR spectrum of **1** revealed the presence of two tertiary methyl groups (s, δ_H_ 2.01, 1.97, 2 × 3H), a methoxy group (s, δ_H_ 3.91, OCH_3_), three quinoid protons centered at d, δ_H_ 6.70, *J* = 10 Hz, H-2; d, 6.89, *J* = 10 Hz, H-3; s, 6.07, H-7′), and two aromatic protons (s, δ_H_ 7.27, H-8; s, 7.64, H-2′). The ^13^C-NMR (BB and DEPT) spectra (**Table 1**) exhibited signals of 23 carbons: two methyl carbons, one methoxy carbon, and 20 carbons for two naphthoquinone units. The quinoid protons resonated as a doublet at δ_H_ 6.70 (H-2) and 6.89 (H-3), showing HMBC correlations with δ_C_ 184.8 (C-1) and 190.3 (C-4). The aromatic proton (s, δ_H_ 7.27) exhibited correlations with δ_C_ 129.4 (C-9) and 114.1 (C-10), while that at s, δ_H_ 7.64 showed correlations with δ_C_ 158.1 (C-1′), 179.5 (C-8′), and 108.6 (C-9′). All the assignments were made with the help of HMBC correlations, as shown in **Figure 2**.

**Table 1:** ^13^C-NMR and ^1^H-NMR spectral data of compound 1.

Carbon no.	δ_C_	δ_H_ (mult, *J*, Hz)
1	184.8	–
2	140.1	6.70, d, (*J* = 10 Hz)
3	137.7	6.89, d, (*J* = 10 Hz)
4	190.3	–
5	161.8	–
6	144.0	–
7	145.5	–
8	125.7	7.27, s
9	129.4	–
10	114.1	–
11	20.6	2.01, s
12-OCH_3_	56.7	3.91, s
1′	158.1	7.64, s
2′	109.4	7.64, s
3′	139.5	–
4′	161.1	–
5′	190.6	–
6′	139.5	–
7′	109.4	6.07, s
8′	179.5	–
9′	108.6	–
10′	112.0	–
11′	20.2	1.97, s

The spectral data identify compound **1** as 5,4′-dihydroxy-1′-methoxy-6,6′-dimethyl-7,3′-binaphthyl-1,4,5′,8′-tetraone, as a new secondary metabolite.

**FIGURE 3:**
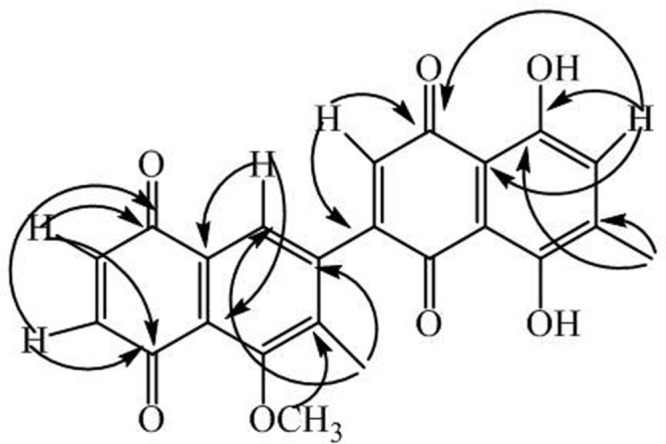
**Key HBMC correlations of compound 2**.

Compound **2** was isolated as a yellow amorphous powder. Its HRMS exhibited the molecular ion peak at *m/z* 404.2100 (calcd: 404.2101), consistent with the molecular formula C_23_H_16_O_7._ The IR spectrum displayed absorption bands at 3560 cm^-1^ for OH stretching, 2925 cm^-1^ for CH stretching, 1644 cm^-1^ for C = O stretching and 1603 cm^-1^ for aromatic proton stretching. The UV spectrum exhibited absorption peaks at 253, 299, and 432 nm. The protons and carbons were assigned out by HMBC, HMQC, ^1^H-^1^H-COSY, and *J*-resolved experiments (**Table 2**). The ^1^H-NMR spectrum of compound **2** showed the presence of two tertiary methyl groups (s, δ_H_ 1.98 and 1.97), one methoxy group (s, δ_H_ 3.90, OCH_3_), three quinoid protons (d, δ_H_ 6.70, *J* = 10.5 Hz, H-2; d, δ_H_ 6.89, *J* = 10.5 Hz, H-3; s, δ_H_ 6.42, H-7′) two aromatic protons (s, δ_H_ 7.27, H-8, and s, 7.59, H-2′). The ^13^C-NMR (BB and DEPT) spectra (**Table 2**) exhibited 23 carbon signals for two methyl carbons, one methoxy carbon, and 20 carbons for two naphthoquinone moieties. The quinoid protons at δ_H_ 6.70 (H-2) and 6.70 (H-3) showed strong HMBC correlations with δ_C_ 184.6 (C-1) and 190.3 (C-4), respectively. The aromatic proton centered at δ_H_ 7.27 (H-8) revealed correlations with δ_C_ 130.4 (C-9) and 114.1 (C-9′), while the quinoid proton at δ_H_ 7.59 (H-2′) showed HMBC correlations with δ_C_ 184.1 (C-1) and 137.6 (C-3). The aromatic proton at δ_H_ 6.42 (H-7′) exhibited correlations with δ_C_ 159.1 (C-8′), 114.1 (C-9′), and 184.1 (C-1). All substituents were assigned with the help of HMBC correlations as shown in **Figure 3**. Compound **2** was characterized on the basis of the spectral data as 5′,8′-dihydroxy-5-methoxy-6,6′-dimethyl-7,3′-binaphthyl-1,4,1′,4′-tetraone.

**Table 2:** ^13^C-NMR and ^1^H-NMR spectral data of compound 2.

Carbon no.	δ_C_	δ_H_ (mult, *J*, Hz)
1	184.6	–
2	140.1	6.70, d, (*J* = 10.5 Hz)
3	137.6	6.70, d, (*J* = 10.5 Hz)
4	190.3	–
5	161.9	–
6	146.1	–
7	148.1	–
8	121.7	7.27, s
9	130.4	–
10	114.1	–
11	20.6	1.98, s
12-OCH_3_	56.6	3.90, s
1′	184.1	–
2′	125.6	7.59, s
3′	137.6	–
4′	190.6	–
5′	161.1	–
6′	148.1	–
7′	110.3	6.42, s
8′	159.1	–
9′	114.1	–
10′	118.5	–
11′	20.5	1.97, s

**FIGURE 4:**
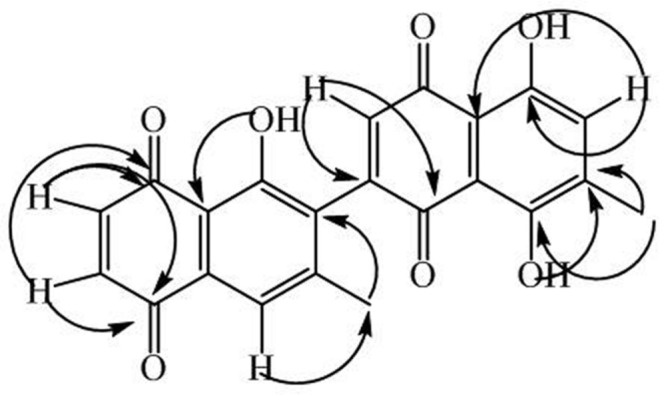
**Key HMBC correlations of compound 3**.

Compound **3** was purified as a yellow amorphous powder. Its HRMS exhibited the molecular ions peak at *m/z* 390.0001 (calcd. 390.0007), consistent with the molecular formula C_22_H_14_O_7._ The IR spectrum displayed absorption bands at 3550 cm^-1^ (OH stretching), 2988 cm^-1^ (CH stretching), 1642 cm^-1^ (C = O stretching) and 1602 cm^-1^ (CH aromatic stretching). The UV spectrum exhibited absorption peaks at 250, 301, and 436 nm. The protons and carbons were assignment by HMBC, HMQC, ^1^H-^1^H-COSY, and *J*-resolved experiments (**Table 3**). The ^1^H-NMR spectrum of **3** showed proton signals indicating the presence of two tertiary methyl groups resonating at δ_H_ 1.97 (s, H-11) and 2.01 (s, H-11′), three quinoid protons at δ_H_ 6.66 (d, *J* = 10 Hz, H-2), 6.89, (d, *J* = 10 Hz, H-3), and δ_H_ 6.94 (s, H-5), and two aromatic proton singlets centered at δ_H_ 7.55 (H-2′), and δ_H_ 7.27 (H-7′). The ^13^C-NMR (BB and DEPT) spectra (**Table 3**) exhibited the resonances of 22 carbons, identified as two methyl carbons and 20 carbons for two naphthoquinone moieties. The quinoid proton signals (δ_H_ 6.66, H-2; 6.89, H-3 and 7.55, H-2′) showed HMBC (**Figure 4**) correlations with δ_C_ 190.2 (C-1), 195.3 (C-4), 137.6 (C-3′), and 190.1(C-4′), while the aromatic proton signals (δ_H_ 6.94, H-5 and 7.27 H-5) correlated with δ_C_ 24.7 (C-11), 159.0 (C-8′) and 112.0 (C-9′), respectively. On the basis of the spectral data, compound **3** is characterized as 8,5′,8′-trihydroxy-6,6′-dimethyl-7,3′-binaphthyl-1,4,1′,4′-tetraone.

**Table 3:** ^13^C-NMR and ^1^H-NMR spectral data of compound 3.

Carbon no.	δ_C_	δ_H_ (mult, *J*, HZ)
1	190.2	–
2	140.0	6.66, d, (*J* = 10 Hz)
3	139.9	6.89, d, (*J* = 10 Hz)
4	195.3	–
5	121.3	6.94, s
6	149.1	–
7	148.0	–
8	162.0	–
9	130.0	–
10	114.1	–
11-CH_3_	24.7	1.97, s
1′	184.8	–
2′	125.0	7.55, s
3′	137.6	–
4′	190.1	–
5′	161.9	–
6′	146.5	–
7′	125.9	7.27, s
8′	159.0	–
9′	112.0	–
10′	121.7	–
11′-CH_3_	20.7	2.01, s

Dimeric naphthoquinones **1–3** were investigated for their potential properties as MDR eﬄux pump modulators. They were first screened for antiproliferative activity on human *ABCB1* gene transfected mouse lymphoma cell line L5178Y, which specifically overexpresses a membrane-localized transporter (*P*-gp, ABCB1). In order to test the potential clinical application of compounds **1–3**, they were evaluated as concerns the reversal of MDR (Csonka et al., 2013). The antiproliferative effects of compounds **1–3** were determined by the MTT method. Compounds **1** and **2** showed promising antiproliferative potential on the L5178Y mouse T-cell lymphoma cell line, with IC_50_ values of 0.05 ± 0.004 μg/mL and 0.046 ± 0.005 μg/mL, respectively, whereas compound **3** had an IC_50_ value of 0.26 ± 0.01 μg/mL (**Table 4**).

**Table 4:** Antiproliferative effects of compounds 1–3 on the L5178Y mouse T-cell lymphoma cell line.

Compounds	IC_50_ (μg/ml)	SEM
**1**	0.05	0.004
**2**	0.046	0.005
**3**	0.26	0.01

*SEM, standard error of the mean.*

**FIGURE 5:**
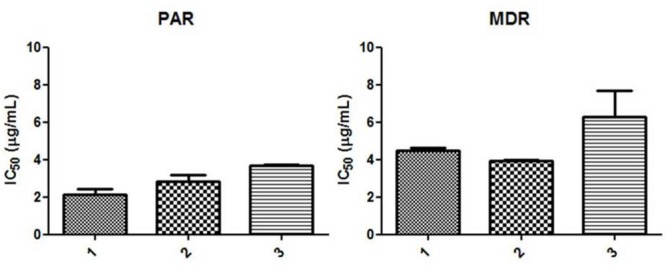
**Cytotoxic effects of dimeric naphthoquinones on PAR and MDR mouse.** T-cell lymphoma cell lines. The mean IC_50_ was calculated on the basis of the results of three independent experiments. SEM, standard error of the mean.

The cytotoxic effects of the dimeric naphthoquinones on the PAR and MDR cell lines were studied. Evaluation of the cytotoxic activities of the compounds revealed that **1** and **3** were the most active against the parental mouse T-lymphoma cell line, with IC_50_ values of <3 μg/mL. Both of these compounds were also active against the MDR cell line (**Figure 5** and **Table 5**).

**Table 5:** Cytotoxic effects of dimeric naphthoquinones on PAR and MDR mouse T-cell lymphoma cell lines.

Compounds	Mean IC_50_ (μg/mL)			
	PAR	SEM	MDR	SEM
**1**	2.15	0.41	4.49	0.17
**2**	2.82	0.50	3.93	0.10
**3**	3.59	0.15	6.29	1.94

*SEM, standard error of the mean.*

Compounds **1–3** were also evaluated for the reversion of the MDR of *ABCB1* gene transfected mouse lymphoma cell line, followed by flow cytometry, which measures intracellular accumulation of rhodamine 123, a fluorescent substrate analog of epirubicin. The fluorescence activity ratio (FAR) value was used to evaluate the ABCB1 transporter modulating potential. When the tested compounds were investigated in concentrations of 0.1 to 1 μg/mL by flow cytometry (**Table 6**), the side scatter count and forward scatter count values increased, indicating that the compounds exerted membrane effects and the granulation of the cytoplasm was increased. The results revealed a special type of toxic effect on the reversal of MDR at toxic doses of 1 μg/mL. The FAR of compounds **1–3** differed: compound **1** proved to be a very effective MDR modulator, while compounds **2** and **3** did not exhibit significant effects in a short-time experiment. Verapamil, a calcium channel blocker and chemosensitizer, was used as a positive control. The results relating to MDR reversal activity in the current investigation are presented in **Table 6**. On MDR mouse lymphoma cells, compounds **1–3** were screened in two concentrations (1 and 0.1 μg/mL). Compound **1** was a fairly moderate modulator of the eﬄux pump activity (FAR = 1.72 at 1 μg/mL and 10.63 at 0.1 μg/mL), while compounds **2** (FAR = 1.1 at 1 μg/mL and 1.3 at 0.1 μg/ml) and **3** (FAR = 1.15 at 1 μg/mL and 0.84 at 0.1 μg/mL) were somewhat weaker (**Table 6**).

**Table 6:** Effects of compounds 1–3 on the reversal of multidrug resistance in mouse lymphoma cells in the presence of low doses (0.1–1 μg/mL).

Compounds	Concentration (μg/mL)	FSC	SSC	FL-1	FAR	MF-1
**Verapamil**	10	1993	613	10	10.5	12
**1**	0.1	2003	606	1.63	1.72	0.673
	1	1982	619	10.1	10.63	17.2
**2**	0.1	2008	609	1.05	1.1	0.806
	1	2024	582	1.24	1.3	0.698
**3**	0.1	2029	606	1.1	1.15	0.806
	1	2013	636	0.801	0.84	0.673
**DMSO**	2% v/v	2108	604	0.782	0.82	0.604

*FSC, forward scatter count of cells in the samples (cell size ratio); SSC, side scatter count of cells in the samples; FL-1, mean fluorescence intensity of the cells; FAR, fluorescence activity ratio; MF-1, maximum fluorescence intensity.*

The effects of the tested compounds on the activity of doxorubicin were observed to vary from slight antagonism to antagonism as shown in **Table 7**.

**Table 7:** Types of interaction between dimeric naphthoquinone derivatives and doxorubicin in MDR mouse T-cell lymphoma cell line.

Compounds	Ratio	ED50	CI	Interaction
**1**	1.724:25 1.724:25 0.862:50^∗^	1.85494 1,77987 1,66629	1.302	Moderate antagonism
**2**	6.897:25^∗^ 3.448:50 0.862:50	1.24794 1.65175 1.66558	1.175	Slight antagonism
**3**	6.897:50 3.448:50 3.448:100^∗^	2.36396 2.49152 1.80217	1.459	Antagonism

*CI, combination index. ^∗^Best ratio.*

The tested dimeric naphthoquinones **1–3** display several structural differences, mainly relating to the positions of the aromatic linkages. Compound **3** differences from compounds **1** and **2** in containing an extra hydroxyl group rather than a methoxy substituent. It is clear that the joint presence of methoxy and hydroxy groups enhanced the activity (**Table 4**). The positions of quinone moieties influenced the eﬄux pump activity, as demonstrated by the FAR values in **Table 6**.

## Conclusion

Three new dimeric naphthoquinones (**1–3**) isolated from the CHCl_3_-soluble fraction of the roots of *D. lotus*, led to the reversal of MDR and exerted antiproliferative activity. They also exhibited promising antiproliferative effects in a dose-dependent manner on two cancer cell lines. This discovery strengthens our belief in the indigenous knowledge of traditional health cures against cancer diseases. The results suggest that research on naphthoquinone derivatives could possibly lead to the discovery of potent anticancer agents.

## Author Contributions

GU and AK were project supervisor. AR was performed isolation of compounds. BS and BA were gave the project idea. JM, AC, and DS were performed the activities of compounds. UF and AK involved in the useful discussion and participated in manuscript writing. All authors read and approved the final manuscript.

## Conflict of Interest Statement

The authors declare that the research was conducted in the absence of any commercial or financial relationships that could be construed as a potential conflict of interest.

## Footnotes

1 www.graphpad.com

2 www.graphpad.com

3 www.flowjo.com

4 http://www.combosyn.com
